# Streptolysin S induces mitochondrial damage and macrophage death through inhibiting degradation of glycogen synthase kinase-3β in *Streptococcus pyogenes* infection

**DOI:** 10.1038/s41598-019-41853-3

**Published:** 2019-03-29

**Authors:** Nina Tsao, Chih-Feng Kuo, Miao-Hui Cheng, Wei-Chen Lin, Chiou-Feng Lin, Yee-Shin Lin

**Affiliations:** 1 Department of Medical Laboratory Science, College of Medicine, I-Shou University, Kaohsiung City, Taiwan; 2 Department of Biological Science and Technology, College of Medicine, I-Shou University, Kaohsiung City, Taiwan; 3 Department of Nursing, College of Medicine, I-Shou University, Kaohsiung City, Taiwan; 4 Department of Microbiology and Immunology, School of Medicine, College of Medicine, Taipei Medical University, Taipei, Taiwan; 5 Department of Microbiology and Immunology, College of Medicine, National Cheng Kung University, Tainan, Taiwan; 6 Center of Infectious Disease and Signaling Research, National Cheng Kung University, Tainan, Taiwan

## Abstract

Group A *Streptococcus* (GAS) infection is associated with a variety of human diseases. Previous studies indicate GAS infection leads to RAW264.7 cell death, but the mechanism is unclear. Here, analyzing the timing of reactive oxygen species (ROS) production and using mitochondrial ROS scavenger, we found the wild type GAS-induced RAW264.7 cell death was associated with mitochondrial ROS. The wild type GAS infection could activate glycogen synthase kinase-3β (GSK-3β). Inhibition of GSK-3β activity by lithium chloride or decreasing GSK-3β expression by lentivirus-mediated short hairpin RNA for GSK-3β could not only decrease the wild type GAS-induced mitochondrial ROS generation, mitochondria damage and cell death, but also reduced GAS intracellular replication. Streptolysin S (SLS), a GAS toxin, played the important role on GAS-induced macrophage death. Compared to the wild type GAS with its isogenic *sagB* mutant (SLS mutant)-infected macrophages, we found *sagB* mutant infection caused less mitochondrial ROS generation and cell death than those of the wild type GAS-infected ones. Furthermore, the *sagB* mutant, but not the wild type or the *sag*B-complementary mutant, could induce GSK-3β degradation via a proteasome-dependent pathway. These results suggest that a new mechanism of SLS-induced macrophage death was through inhibiting GSK-3β degradation and further enhancing mitochondrial damage.

## Introduction

Group A *Streptococcus* (GAS; *Streptococcus pyogenes*) infection leads to a wide spectrum of human diseases from noninvasive impetigo to invasive necrotizing fasciitis. GAS produces several virulence factors, such as M protein, fibronectin-binding proteins, streptococcal pyrogenic exotoxin B (SPE B), Ig-degrading enzyme, and leukocidal toxins which hinder the host immune system-mediated opsonophagocytosis^1^. Two leukocidal toxins are produced by GAS, one is oxygen-labile streptolysin O (SLO) and the other is oxygen-stable streptolysin S (SLS). SLO is a well-documented cytolysin with cell-lytic and tissue-destructive activity, and is involved in severe tissue damage induced by GAS infection. The anti-SLO titer is used as an indicator of streptococcal infection because SLO is a potent immunogen to humans^2^. SLS is the other potent cytolysin with a wide range of targets, but the immunogenicity of SLS is lower compared with SLO. Anti-SLS antibody induced by immunizing with the SLS peptide, coupled to keyhole limpet hemocyanin or with the SLS epitope-contained recombinant protein, can reduce the SLS-mediated hemolysis and mortality of GAS-infected mice^3,4^. The cytotoxic effects of SLS have been reported in different types of cells, in which it mediated apoptosis, oncosis, and activation of the proinflammatory signaling pathways during the invasive GAS infection^5–9^. However, the detailed mechanisms involved in SLS-induced cell death are not well-understood.

Glycogen synthesis kinase-3β (GSK-3β), an important regulator of multiple cellular functions, controls metabolism, survival signaling and death signaling of cells^10^. GSK-3β mediates the opening of mitochondrial permeability transition pores (mPTP) and induces the change of mitochondrial membrane potential (ΔΨm)^11,12^. The activity of GSK-3β is also associated with bacterial internalization and pathogenesis of infection^13–15^. GSK-3β plays an essential role in regulating inflammatory responses, in which it may either enhance or inhibit the inflammatory response, depending on different pathogens, virulence factors, and the type of the infected cells^16^.

SLS is encoded by the streptococcal *sagA* gene and posttranslationally modified by heterocycle-forming synthetase proteins SagB, SagC and SagD to produce a mature pore-forming streptolysin S^5,17^. SagB serves as a dehydrogenase which cooperates with cyclodehydratase to catalyze heterocycle formation and maturation of streptolysin S with cytolytic activity^18^. Our previous study indicates that mutation of *sagB* attenuates GAS-induced production of proinflammatory cytokines and mice death. Furthermore, the cell death percentage of the *sagB* mutant-infected phagocytes is lower than that of the wild type GAS-infected ones^19^. In this study, we found the wild type GAS-induced macrophage death was associated with GSK-3β activation, mitochondrial ROS production and mitochondrial damage. Moreover, we suggest a new mechanism of SLS-induced cell death through inhibiting a proteasome-dependent degradation of GSK-3β and further enhancing mitochondrial damage and cell death.

## Results

### Mitochondrial reactive oxygen species (ROS) mediated cell death in the wild type GAS-infected RAW264.7 cells

**Figure 1:**
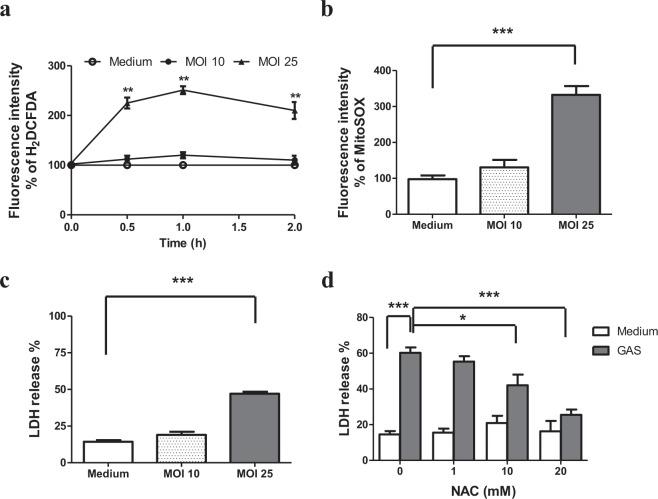
ROS-mediated cytotoxicity in the GAS-infected RAW264.7 cells. RAW264.7 cells were infected with the wild type GAS at MOI 10 or 25. The levels of ROS were measured by carboxy-H_2_DCFDA (**a**) at different times post-infection or by MitoSOX (**b**) staining at 3 h post-infection. Fluorescence intensity % was shown and expressed as described in Materials and Methods. Results are represented as mean ± standard deviation (SD). **P < 0.01, ***P < 0.001 compared with medium only group (One-way ANOVA test followed by Tukey’s test; n = 4). The culture supernatants of RAW264.7 cells infected with the GAS at MOI 10 or 25 (**c**), and the culture supernatants of RAW264.7 cells infected with GAS at a MOI of 25 in the presence of different concentrations of NAC (**d**) were collected at 18 h post-infection and then measured by LDH detection kit. In (**c**), LDH release % was shown and expressed as the mean ± SD, as described in Materials and Methods. ***P < 0.001 compared with medium only group (One-way ANOVA test followed by Tukey’s test; n = 4). In (**d**), LDH release % was shown and expressed as the mean ± SD, as described in Materials and Methods. ***P < 0.001 compared with GAS-infected group versus medium only group. (One-way ANOVA test followed by Tukey’s test; n = 4).***P < 0.001 compared with 20 mM NAC- treated group versus GAS-infected group (One-way ANOVA test followed by Tukey’s test; n = 4). *P < 0.05 compared with 10 mM NAC- treated group versus GAS-infected group (One-way ANOVA test followed by Tukey’s test; n = 4).

The intracellular H_2_O_2_ and mitochondrial ROS levels of the RAW264.7 cells infected with GAS at multiplicity of infection (MOI) of 10 or 25 were determined using membrane-permeant 6-carboxy-2′,7′-dichlorodihydrofluorescein diacetate (carboxy-H_2_DCFDA) and MitoSOX reagents respectively. As shown in Fig. 1a, the level of intracellular H_2_O_2_ on GAS-infected RAW264.7 cells, when compared with the uninfected control cells, rapidly increased and reached a peak at 1 h post-infection, followed by a decline. The increased ratio of H_2_O_2_ on RAW264.7 cells infected with GAS at MOI of 25 was significantly higher than that of the cells infected with GAS at MOI of 10 (251 ± 7.85% versus 115 ± 4.16%) at 1 h post-infection. However, the mitochondrial ROS of RAW264.7 cells significantly increased beginning at 3 h post-infection of wild-type GAS (Fig. 1b) and the ROS level was sustained until 18 h post-infection. The increased level of mitochondrial ROS was dependent on MOI of GAS (332 ± 24.5% at MOI 25 versus 131 ± 2.92% at MOI 10) (Fig. 1b). For the examination into the cell death by LDH release assay, we noted that GAS at MOI 25 could induce significant cell death at 18 h post-infection (Fig. 1c). To examine the role of the ROS increase on GAS-induced cell death, we measured the levels of LDH release from the wild type GAS-infected RAW264.7 cells in the presence of varying concentrations of ROS scavenger, N-acetyl cysteine (NAC). As shown in Fig. 1d, the LDH release from the GAS-infected cells was significantly decreased in the presence of 10 mM and 20 mM NAC. This result indicates that a decrease of ROS levels by NAC could prevent cell death of wild type GAS-infected RAW264.7 cells. The ROS sources in wild type GAS-infected cells may be phagosomes or mitochondria. Based on LDH release assay, we found that no significant cell death appeared at 5 h post wild-type GAS infection but cell death was significantly increased at 18 h post wild-type GAS infection (Supplementary Fig. S1), which corresponded with the increase of mitochondrial ROS, but not with early phagosome ROS, after infection. Moreover, the cell death of wild type GAS-infected RAW264.7 cells was significantly decreased in the presence of mitochondria-targeted antioxidant Mito-Tempo (Supplementary Fig. S2). These results indicate that the wild type GAS-mediated cytotoxicity was associated with the elevation of mitochondrial ROS.

### Inhibition of GSK-3β prevented the elevations of mitochondrial ROS and cell death in the wild type GAS-infected RAW264.7cells

**Figure 2:**
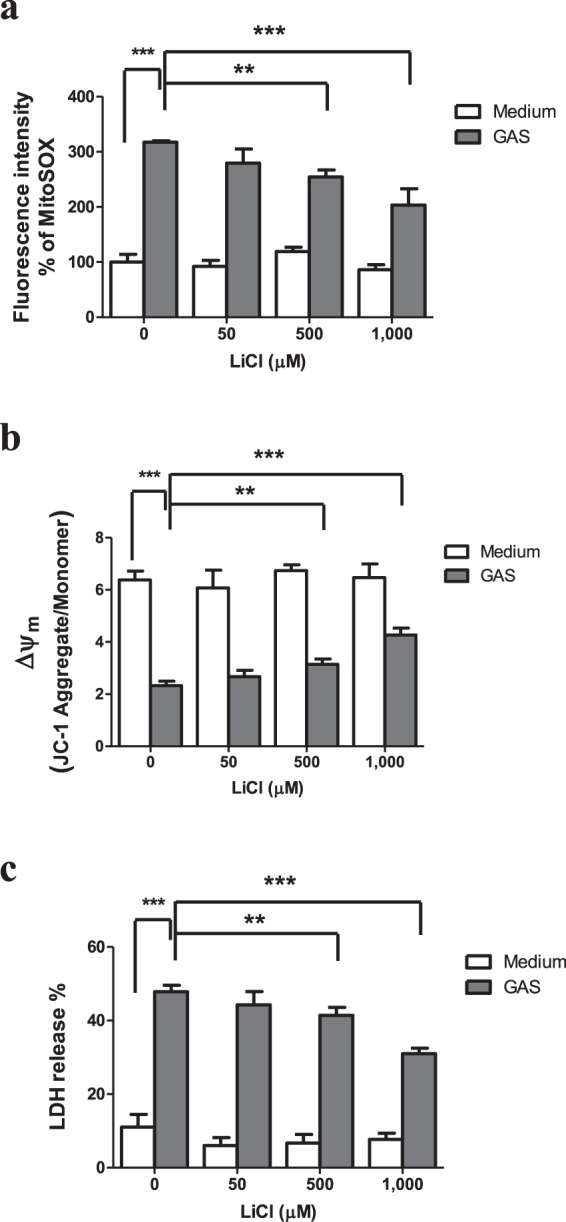
LiCl inhibited the mitochondrial ROS production and prevented the loss of Δψm and cell death in the GAS-infected RAW264.7 cells. RAW264.7 cells were infected with the wild type GAS for 1 h at a MOI of 25 in the presence of different concentrations of LiCl. Mitochondrial ROS and Δψm of the GAS-infected cells were measured by MitoSOX and JC-1 respectively at 3 h post-infection. (**a**) Mitochondrial ROS production in the GAS-infected RAW264.7 cells was inhibited by LiCl in a dose-dependent manner. Fluorescence intensity % was shown and expressed as described in Materials and Methods. Results are represented as mean ± SD. In LiCl-treated groups, **P < 0.01, ***P < 0.001 compared with the GAS-infected group. In the GAS-infected group, ***P < 0.001 compared with medium only (without GAS) group (One-way ANOVA test followed by Tukey’s test; n = 4). (**b**) The change of Δψm in GAS-infected cells was inhibited by LiCl. The level of Δψm in RAW264.7 cells was detected by JC-1 as described in Materials and Methods. In LiCl-treated groups, **P < 0.01, ***P < 0.001 compared with the GAS-infected group. In the GAS-infected group, ***P < 0.001 compared with medium only (without GAS) group (One-way ANOVA test followed by Tukey’s test; n = 4). (**c**) The LDH release of GAS-infected cells was dose-dependently inhibited by LiCl. The culture supernatants of GAS-infected RAW264.7 cells were collected at 18 h post-infection and then measured by LDH detection kit. LDH release % was shown and expressed as the mean ± SD, as described in Materials and Methods. In LiCl-treated groups, **P < 0.01, ***P < 0.001 compared with GAS-infected group. In the GAS-infected group, ***P < 0.001 compared with medium only (without GAS) group (One-way ANOVA test followed by Tukey’s test; n = 4).

Reports indicate that mitochondrial ROS induction is associated with activation of GSK-3β^11,12,20^. GSK-3β regulates the opening of mPTP and the change of ΔΨm^11,12,20^. Inactivation of GSK-3β facilitates recovery of ΔΨm and reduces the oxidant stress-induced cell death^11^. We examined effects of GSK-3β inhibitor lithium chloride (LiCl) on rotenone, a mitochondrial ROS generator, -treated RAW264.7 cells, and found that rotenone-induced the generation of mitochondrial ROS (Supplementary Fig. S3a) and cell death (Supplementary Fig. S3b) of RAW264.7 cells were dose-dependently inhibited by LiCl. In this study, we further tested the mitochondrial ROS levels and change of ΔΨm of the wild type GAS-infected RAW264.7 cells in the presence of varying concentrations of LiCl. LiCl had no antimicrobial activity with respect to *in vitro* growth of GAS (Supplementary Fig. S4). LiCl (50~1,000 μM) did not cause the cell death of RAW264.7 cells (Supplementary Fig. S3b & Fig. 2c). As shown in Fig. 2, LiCl effectively reduced the generation of mitochondrial ROS and prevented the loss of ΔΨm in the wild type GAS-infected RAW264.7 cells (Fig. 2a,b). Furthermore, LiCl treatment dose-dependently decreased the LDH release from the wild type GAS-infected RAW264.7 cells (Fig. 2c). These results suggest that activation of GSK-3β might contribute to the mitochondrial ROS generation, the change of ΔΨm, and further promote cell death in the wild type GAS-infected RAW264.7 cells.

**Figure 3:**
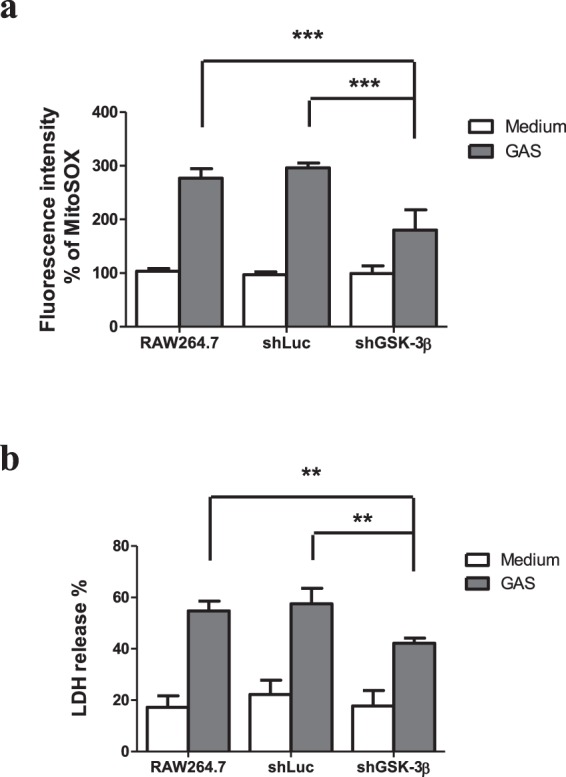
Knockdown of GSK-3β attenuated mitochondrial ROS production and cell death of the GAS-infected RAW264.7 cells. The expression of GSK-3β was silenced in RAW264.7 cells using lentiviral-based shRNA (shGSK-3β) construct and the luciferase shRNA construct was using as negative control. The transfected cells and RAW264.7 cells (without transfection) were infected with GAS at a MOI of 25 as described in Materials and Methods. (**a**) Mitochondrial ROS levels were measured by MitoSOX staining at 3 h post GAS infection. Fluorescence intensity % was shown and expressed as described in Materials and Methods. Results are represented as mean ± SD. In GAS-infected groups, ***P < 0.001 compared with shGSK-3β knockdown group versus negative control group (One-way ANOVA test followed by Tukey’s test; n = 4) or versus the group of RAW264.7 cells (One-way ANOVA test followed by Tukey’s test; n = 4). (**b**) The GAS-mediated cell deaths were measured by LDH release at 18 h post GAS infection, as described in Materials and Methods. **P < 0.01 compared with shGSK-3β knockdown group versus negative control group (One-way ANOVA test followed by Tukey’s test; n = 4) or versus the group of RAW264.7 cells (One-way ANOVA test followed by Tukey’s test; n = 4).

Several intracellular action targets of LiCl such as GSK-3, inositol monophosphatase, phosophomonoesterase, and phosphoglucomutase have been identified^21,22^. In order to confirm that wild type GAS-induced mitochondrial ROS production and cell death was through GSK-3β activation, we used the lentivirus-mediated short hairpin RNA (shRNA) for GSK-3β to knock down GSK-3β expression. Western blotting revealed the levels of total GSK-3β proteins in the shGSK-3β-transfected RAW264.7 cells were reduced compared with the shLuc-transfected control cells (Supplementary Fig. S5). Using rotenone to treat RAW264.7 cells, shLuc-RAW264.7 cells, or shGSK-3β-RAW264.7 cells, we found that rotenone-induced the increases of both mitochondrial ROS and cell death were significantly inhibited in the shGSK-3β-RAW264.7 cells compared with those of rotenone-treated RAW264.7 cells or shLuc-RAW264.7 cells (Supplementary Fig. S6a,b). To further examine the wild type GAS-infected cells, the level of mitochondrial ROS in the wild type GAS-infected RAW264.7 cells or shLuc-RAW264.7 cells was significantly higher than that of the GAS-infected shGSK-3β-RAW264.7cells (Fig. 3a). Analysis of the confocal microscope also revealed that the wild type GAS-infected shGSK-3β-RAW264.7 cells showed weaker MitoSOX staining compared with the wild type GAS-infected RAW264.7cells and shLuc-RAW264.7 cells (Supplementary Fig. S7). Furthermore, the level of LDH release from the wild type GAS-infected shGSK-3β cells was lower than that of the GAS-infected RAW264.7 cells or shLuc-RAW264.7 cells at 18 h after GAS infection (Fig. 3b). These results indicate that reduction of the cellular GSK-3β amount could decrease mitochondrial ROS and cell death of the wild type GAS-infected RAW264.7 cells.

### Inhibition of GSK-3β reduced intracellular bacterial load of the wild type GAS-infected RAW264.7 cells

**Figure 4:**
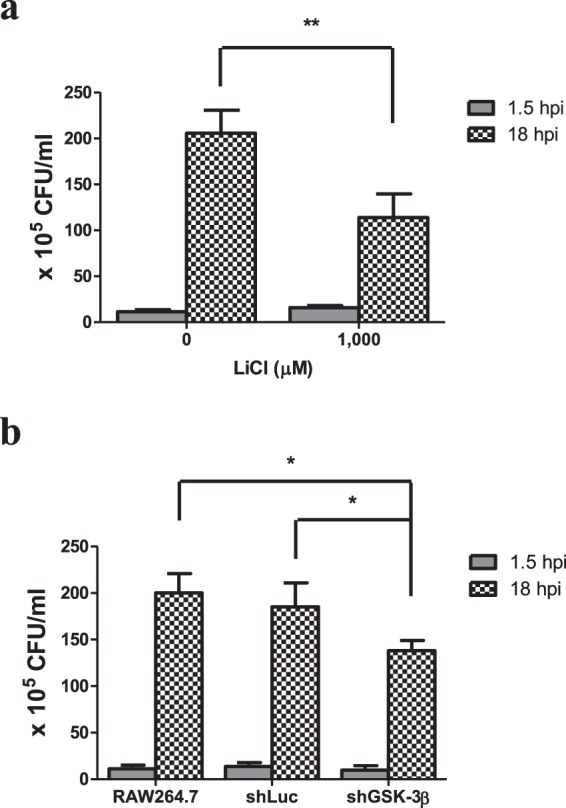
Inhibition of GSK-3β by LiCl or knockdown of GSK-3β reduced the bacterial loads of the GAS-infected RAW264.7 cells. RAW264.7 cells that pretreated with 1,000 μM of LiCl (**a**) or transfected with the shLuc construct or shGSK-3β construct (**b**) were infected with GAS at MOI of 25. The number of remnant bacteria in each group was determined by plate count at 1.5 h or 18 h post GAS infection and shown as described in Materials and Methods. Results are represented as mean ± SD. In (**a**), **P < 0.01 compared with non-LiCl group (Unpaired Student’s *t*-test; n = 4). In (**b**), *P < 0.05 compared with shGSK-3β knockdown group versus negative control group (One-way ANOVA test followed by Tukey’s test; n = 4) or versus the group of RAW264.7 cells (One-way ANOVA test followed by Tukey’s test; n = 4).

To test whether GSK-3β inhibition influenced intracellular GAS replication, we estimated the amount of remaining bacteria in the wild type GAS-infected RAW264.7 cells with or without LiCl at 1.5 h or 18 h post-infection using a plate counting assay. At the initial time point (1.5 h) post-infection, the numbers of intracellular bacteria were similar between the LiCl-treated group and the non-LiCl-treated one, indicating that treatment of LiCl could not interfere with the GAS uptake into the cells. However, at 18 h post-infection, treatment of LiCl significantly decreased intracellular bacterial loads in the wild type GAS-infected RAW264.7 cells (Fig. 4a). We further tested the bacterial clearance of the shGSK-3β-RAW264.7 cells. As shown in Fig. 4b, the GAS uptake was not significantly different among the groups of the wild type GAS-infected RAW264.7 cells, shLuc-RAW264.7 cells, and shGSK-3β-RAW264.7 cells at 1.5 h post-infection. However, the intracellular bacterial load of the wild type GAS-infected shGSK-3β-RAW264.7 cells was reduced compared with that of the GAS-infected RAW264.7 cells or the GAS-infected shLuc-RAW264.7 cells at 18 h post-infection (Fig. 4b). These results indicate that both inhibition of GSK-3β activity and reducing GSK-3β expression decreased intracellular bacterial loads in the wild type GAS-infected RAW264.7 cells.

### Mitochondrial ROS level and the change of ΔΨm were reduced in the *sagB* mutant-infected RAW264.7 cells

**Figure 5:**
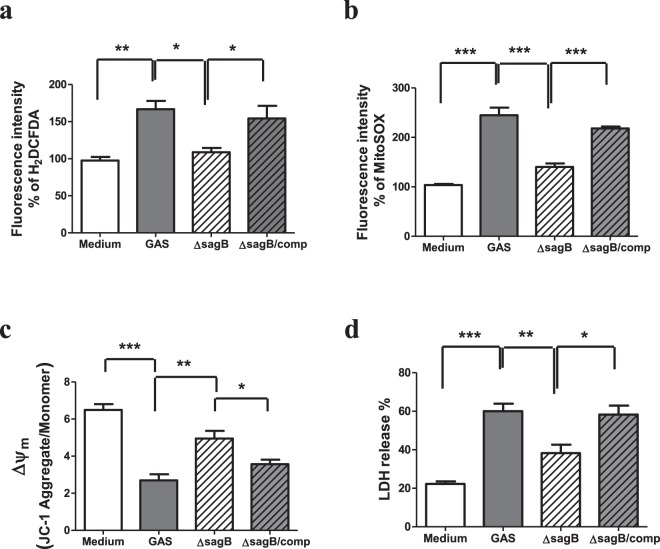
The *sagB* mutation decreased mitochondrial ROS production and prevented mitochondrial dysfunction and cell death in the GAS-infected RAW264.7 cells. RAW264.7 cells were infected with the wild type GAS, isogenic *sagB* mutant or *sagB-* complementary mutant at a MOI of 25 as described in Materials and Methods. (**a**) The levels of ROS were measured by carboxy-H_2_DCFDA at 1 h post GAS infection, as described in Materials and Methods. Results are represented as mean ± SD. **P < 0.01 compared with medium only group. *P < 0.05 compared with *sagB* mutant group versus GAS group or versus *sagB-*complementary group (One-way ANOVA test followed by Tukey’s test; n = 4). Mitochondrial ROS levels and Δψm were measured by MitoSOX (**b**) and JC-1 (**c**) at 3 h post-infection respectively, as described in Materials and Methods. Results are represented as mean ± SD. In (**b**), ***P < 0.001 compared with GAS-infected group versus medium only group. ***P < 0.001 compared with *sagB* mutant group versus GAS group or versus *sagB-*complementary group (One-way ANOVA test followed by Tukey’s test; n = 4). In (**c**), ***P < 0.001 compared with GAS-infected group versus medium only group. **P < 0.01 compared with *sagB* mutant group versus GAS group. *P < 0.05 compared with *sagB* mutant group versus *sagB-*complementary group (One-way ANOVA test followed by Tukey’s test; n = 4). (**d**) The cell death of the wild type, the isogenic *sagB* mutant or *sagB-*complementary mutant-infected cells were determined by LDH release at 18 h post-infection. ***P < 0.001 compared with GAS-infected group versus medium only group. **P < 0.01 compared with *sagB* mutant group versus GAS group. *P < 0.05 compared with *sagB* mutant group versus *sagB-*complementary group (One-way ANOVA test followed by Tukey’s test; n = 4).

Our previous study indicates that SLS and SPE B have a synergistic effect on the GAS invasive infection, and both are involved in GAS-mediated cell death^19^. SPE B has been reported to induce mitochondrial dysfunction and enhance caspase-mediated cell death^23^. However, the mechanism of SLS-mediated cell death is unclear. In this study, we examined whether the levels of mitochondrial ROS and the change of ΔΨm were affected by SLS in the GAS-infected RAW264.7 cells. As shown in Fig. 5a,b, the intracellular ROS levels of the *sagB* mutant-infected cells were significantly lower than those of the wild type GAS or the *sag*B-complementary mutant-infected cells. Analysis of the confocal microscope also revealed that the *sagB* mutant-infected RAW264.7cells showed weaker MitoSOX staining when compared with the wild type GAS or the *sag*B-complementary mutant-infected cells (Supplementary Fig. S8). Moreover, the loss of ΔΨm in the *sagB* mutant-infected cells was significantly reduced compared to that of the wild type GAS or the *sag*B-complementary mutant-infected RAW264.7 cells (Fig. 5c). Using the LDH release assay to detect cell death also revealed that the cell death was significantly decreased in the *sagB* isogenic mutant-infected RAW264.7 cells when compared with that of the wild type GAS or the *sag*B-complementary mutant-infected cells (Fig. 5d). These results indicate that SLS played an important role in causing the mitochondrial damage and cell death in the GAS-infected RAW264.7 cells.

**Figure 6:**
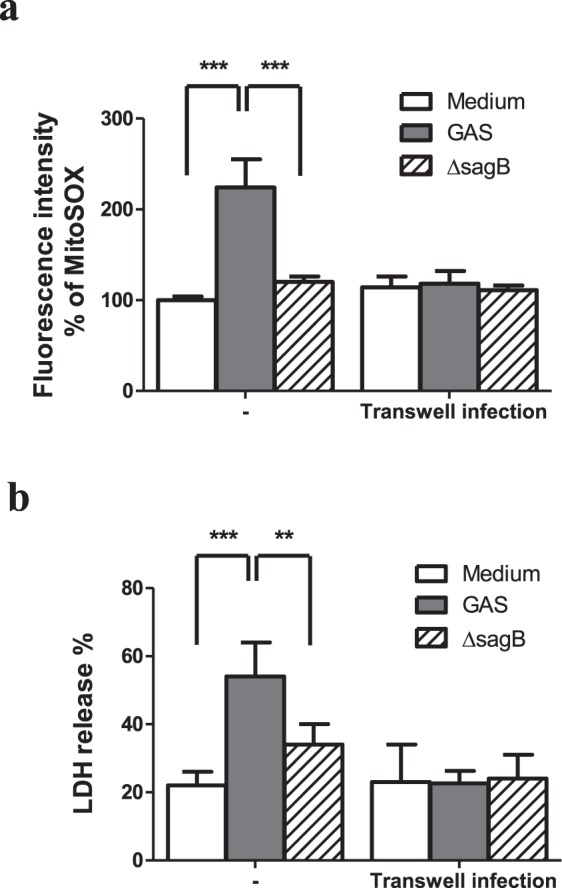
Loss of contact between GAS and RAW264.7 cells reduced mitochondrial ROS production and cell death in the GAS-infected RAW264.7 cells. RAW264.7 cells were infected with the wild type GAS or its isogenic *sagB* mutant, which was added in culture medium or within the hanging insert with 0.4 μm membrane, at a MOI of 25 as described in Materials and Methods. (**a**) Mitochondrial ROS levels were measured by MitoSOX at 3 h post-infection, as described in Materials and Methods. Results are represented as mean ± SD. ***P < 0.001 compared with GAS-infected group versus medium only group or versus *sagB* mutant-infected group (Two-way ANOVA test followed by Bonferroni test; n = 4). (**b**) The cell death of the wild type or the isogenic *sagB* mutant-infected cells were determined by LDH release at 18 h post-infection. Results are represented as mean ± SD. ***P < 0.001 compared with GAS-infected group versus medium only group. **P < 0.01 compared with *sagB* mutant-infected group versus GAS-infected group (Two-way ANOVA test followed by Bonferroni test; n = 4).

To examine whether SLS-mediated the mitochondrial damage and cell death needed the direct contact between GAS and RAW264.7 cells and further GAS internalization, we used the transwell-based infection assay. As shown in Fig. 6, the wild type GAS or the *sag*B mutant-induced the increase of mitochondrial ROS and cell death in RAW264.7 cells vanished when infection of GAS using the culture plate insert with 0.4 μm pores. Based on the confocal microscopic observations, the bacteria were obviously seen in the cytoplasmic space of infected cells by 4′, 6-diamidino-2-phenylindole (DAPI) staining regardless of infection with the wild type, *sag*B mutant or the *sag*B-complementary mutant GAS (Supplementary Fig. S8). These results indicate that the direct contact between GAS and RAW264.7 cells and further bacterial internalization were necessary for GAS to induce the increase of both mitochondrial ROS and cell death in RAW264.7 cells.

### The *sagB* mutant GAS induced a proteasome-mediated GSK-3β degradation in the GAS-infected RAW264.7 cells

**Figure 7:**
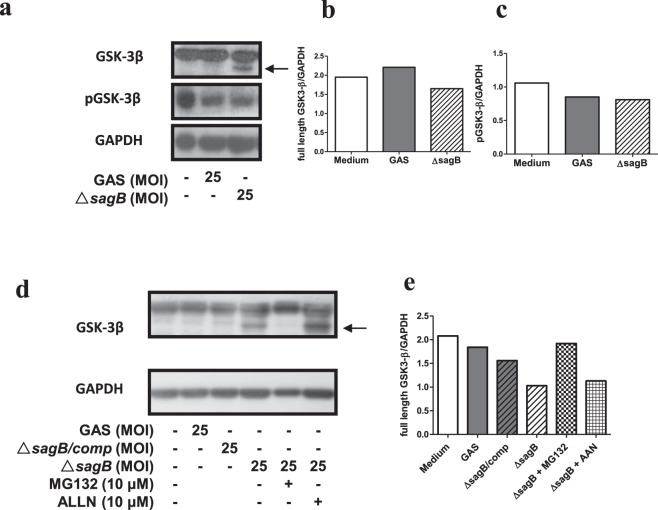
The *sagB* mutation enhanced the proteasome-mediated GSK-3 β degradation in GAS-infected RAW264.7 cells. RAW264.7 cells were infected with the wild type GAS or isogenic *sagB* mutants at a MOI of 25 as described in Materials Methods. (**a**) The phosphorylation of GSK-3β at serine 9 (pGSK-3β) and the total GSK-3β protein in GAS-infected cells were detected at 3 h post-infection by Western blotting, as described in Materials and Methods. Representative immunoblots from 3 independent experiments were shown. The black arrow indicates low MW of GSK-3β. The quantitative ratios of full-length GSK-3β relative to GAPDH were determined by ImageJ and shown in (**b**). Displayed blots of GSK-3β and GAPDH are cropped from the same gel, and then immunoblotted with anti-GSK-3β antibody and anti-GAPDH antibody respectively. Full-length blots are presented in Supplementary Fig. S9a,b. The quantitative ratios of pGSK-3β relative to GAPDH were determined by ImageJ and shown in (**c**). Displayed blots of pGSK-3β are cropped from the independent gel. Full-length blots are presented in Supplementary Fig. S9c,d. (**d**) RAW264.7 cells were infected with the wild type, *sag*B mutant, or *sag*B-complementary mutant at a MOI of 25. In some groups, cells were pretreated with 10 μM of MG132 (proteasome inhibitor) or 10 μM of ALLN (calpain inhibitor I) before the *sag*B mutant infection, as described in Materials and Methods. Total GSK-3β protein in GAS-infected cells were detected at 3 h post-infection by Western blotting. GAPDH was used as an internal control. The black arrow indicates low MW of GSK-3β. The quantitative ratios of full-length GSK-3β relative to GAPDH were determined by ImageJ and shown in (**e**). Displayed blots are not cropped from the different gels. Full-length blots are presented in Supplementary Fig. S9e,f.

The mitochondrial dysfunction and the cell death were reduced in the *sagB* mutant-infected RAW264.7 cells when compared with the wild type GAS-infected RAW264.7 cells (Fig. 5). Further testing the activity of GSK-3β in the GAS-infected RAW264.7 cells revealed that a reduction of GSK-3β serine 9 phosphorylation, which was an indication of GSK-3β inactivation, was observed both in the wild type GAS- and the *sagB* mutant-infected RAW264.7 cells at 3 h post-infection. However, a low molecular weight (MW) form of GSK-3β was detected in the *sagB* mutant- but not in the wild type GAS-infected RAW264.7 cells (Fig. 7a). This result indicates that the decreasing serine 9 phosphorylation of GSK-3β in the *sagB* mutant-infected RAW264.7 cells was attributed to the degradation of GSK-3β, but not an indication of GSK-3β activation. Moreover, the low MW GSK-3β was not notable both in the wild type GAS- and the *sag*B-complementary mutant-infected RAW264.7 cells, indicating that GSK-3β was actually activated after wild-type GAS infection (Fig. 7). Treatment with the proteasome inhibitor MG132 (10 μM) inhibited the degradation of GSK-3β in the *sagB* mutant-infected RAW264.7 cells and diminished the low MW GSK-3β. However, the presence of 10 μM of calpain inhibitor I, N-acetyl-Leu-Leu-norleucinal (ALLN), did not prevent the degradation of GSK-3β of the *sagB* mutant-infected RAW264.7 cells (Fig. 7d,e). These results suggest that a proteasome-mediated GSK-3β degradation was induced in RAW264.7 cells by *sagB* mutant GAS infection, and that this prevented *sagB* mutant GAS-induced mitochondrial damage and cell death.

## Discussion

Macrophages are important first-line defense cells in tissues to fight against bacterial infection. The NADPH oxidase complex (NOX) and mitochondria are two major sources of ROS generation in bacteria-infected cells^24,25^. ROS amount and its generation source in cells contribute to cellular regulation, including cell cycle progression, differentiation, migration and cell death^26^. In general, ROS, especially that produced by NOX, plays an important role in phagocytes to kill bacteria in phagosomes^27^. It has also been indicated that ROS produced both by NOX and mitochondria play an important role in enhancing the bacterial killing activity of macrophages^28^. However, the elevation of ROS levels in epithelial cells by mitochondrial NLRX1, a member of the Nod-like receptor family, contributes to chlamydial growth and induces caspase-1 activation^24^. In the present study, we found that GAS infection enhanced ROS production of RAW264.7 cells, and reduction of ROS levels by NAC could increase the cell survival after GAS infection (Fig. 1), suggesting that increased intracellular ROS contributed to cell death. Analysis of ROS generation sources, infection periods, and timing of cell death indicate that mitochondrial ROS, produced beginning 3 h after GAS infection and sustaining until 18 h, played the major role of causing cell death of GAS-infected cells (Figs 1 and 5). Reports have indicated that mitochondrial complex I inhibitor rotenone induces mitochondrial ROS production, endoplasmic reticulum stress, and activation of GSK-3β^20,29^. We also found that the cell death of RAW264.7 cells was significantly increased in the presence of 1 μM rotenone (Supplementary Fig. S3b). Moreover, the cell death of GAS-infected RAW264.7 cells was decreased in the presence of mitochondria-targeted antioxidant Mito-Tempo (Supplementary Fig. S2). These results show that GAS infection mediated the mitochondrial ROS elevation and enhanced the GAS-infected cell death.

In addition to mitochondrial ROS production induced by GAS, we found that H_2_O_2_ production rapidly increased, reaching a peak at 1 h post-infection, subsequently declining, and that the increased level of H_2_O_2_ was dependent on the MOI of GAS (Fig. 1a). Based on LDH release assay, we found GAS-induced cell death appeared at 5 h post-infection even there was no significant difference compared with the medium-only group, and that GAS-induced cell death increased significantly with infection times (Supplementary Fig. S1). Studies have indicated that NOX-derived ROS is involved in GSK-3β activation, and following NF-κB activation as well as tumor necrosis factor-α (TNF-α)-induced cell death in GAS infection^30,31^. Chen *et al*. indicate that GAS infection can activate NOX and GSK-3β beginning at 0.5 h post-infection, and treatment of diphenyleneiodonium, a general inhibitor of NOX, is able to inactivate GSK-3β in GAS-infected RAW264.7 cells^31^. This result suggests that NOX-derived ROS appeared at the early stage of infection plays a role to activate GSK-3β. GSK-3β regulates the opening of mPTP that triggers the loss of ΔΨm and causes cell necrosis^11,12^. In this study, using LiCl and shGSK-3β to decrease GSK-3β activity and its expression, we identified that GSK-3β was involved in the regulation of mitochondrial ROS generation, the changing of ΔΨm, and cell death of GAS-infected RAW264.7 cells (Figs 2 and 3). Decreasing GAS-induced cell death by inhibition of GSK-3β would reduce the intracellular GAS replication of RAW264.7 cells (Figs 2–4). We hypothesize that the early ROS production, which was derived by NOX, might play a role to activate GSK-3β after GAS infection and then drove the subsequent mitochondrial damage and cell death.

Our previous study has shown that mutation of *spe*B or *sag*B attenuates the degrees of skin lesion and mortality of the GAS-infected mice, indicating that SPE B and SLS have synergistic effects on the GAS invasive infection. Moreover, both toxins are involved in GAS-mediated cell death^19^. SPE B has been reported to induce mitochondrial dysfunction and enhance caspase-mediated cell death^23^. However, whether SLS-mediated cell death is through a mitochondria pathway remains unclear. In this study, a *sag*B-dependent elevation of mitochondrial ROS and a change of ΔΨm were observed in the GAS-infected RAW 264.7 cells. A degradation of GSK-3β was displayed in the *sag*B mutant-infected RAW264.7 cells that was associated with the reduction of mitochondria-mediated cell death after GAS infection (Figs 5 and 7). Most cellular proteins are degraded by the ubiquitin proteasome system or the autophagic lysosomal pathway. Studies indicate that truncation of C-terminal GSK-3β by calpain I markedly increases GSK-3β activity and involvement of this mechanism is probably responsible for enhancing GSK-3β activity and the consequent abnormal hyperphosphorylation of tau and neurofibrillary degeneration in Alzheimer’s disease^32^. In the present study, we tested the effect of calpain I inhibitor ALLN and proteasome inhibitor MG132 on the *sagB* mutant infection-mediated GSK-3β degradation. As shown in Fig. 7d,e, treatment with ALLN did not reduce the level of GSK-3β degradation in the *sagB* mutant-infected RAW 264.7 cells, indicating that calpain I might not mediate the *sag*B mutant-induced GSK-3β degradation. These data indirectly indicate that *sag*B mutant-induced GSK-3β degradation may not induce GSK-3β activation. Our Western blot results showed that the proteasome inhibitor MG132 significantly reduced the level of GSK-3β degradation, suggesting that a proteasomal degradation of GSK-3β was induced in the *sagB* mutant-infected RAW264.7 cells (Fig. 7d,e). Failor *et al*. report that serum- and glucocorticoid-induced protein kinase (Sgk) and Akt trigger the glucocorticoid-regulated phosphorylation, ubiquitination, and degradation of GSK-3β at its serine 9 phosphorylation site in dexamethasone-treated epithelial cells. Sgk and Akt phosphorylate serine 9 of GSK-3β, which leads to an ubiquitin-26S proteasome-mediated degradation of GSK-3β in dexamethasone-treated epithelial cells^33^. Our Western blot results also indicate that degradation of Akt were presented both in the wild type GAS- and the *sag*B mutant-infected RAW264.7 cells, with the degradation of Akt being significantly obvious in the *sag*B mutant-infected RAW264.7 cells (unpublished observations). However, the degradation of GSK-3β was only observed in the *sag*B mutant-infected RAW264.7 cells (Fig. 7). These results indicate that the *sag*B mutant-induced GSK-3β degradation might not be through the AKT pathway. Whether the autophagic lysosomal pathway or the ubiquitin proteasome system directly causes the GSK-3β degradation of the *sagB* mutant-infected RAW264.7 cells needs further investigation.

GSK-3β plays an important role during microbial infection, mediating the activation of NF-κB and regulating the expression of proinflammatory cytokines^34–36^. Dextromethorphan enhances bactericidal activity and reduces inflammatory cytokines to prevent sepsis in GAS infection^37^. A recent report indicates that inhibition of NOX-derived ROS production by dextromethorphan attenuates GSK-3β and NF-κB activation, and then reduces nitric oxide, TNF-α, and interleukin-6 production in GAS-infected RAW264.7 cells^31^. Here we found that treatment with GSK-3 inhibitor or knockdown of GSK-3β not only decreased mitochondrial ROS production, mitochondrial damage as well as cell death, but also reduced the intracellular GAS replication of GAS-infected RAW264.7 cells (Figs 2–4). We also demonstrated that SLS-induced cell death of RAW264.7 cells was accomplished through inhibiting GSK-3β degradation and further GSK-3β-mediated mitochondrial damage (Figs 5 and 7). These data emphasized the role of GSK-3β activation on macrophage death during GAS infection. In addition to our findings here, Marchand *et al*. demonstrate that GSK-3β inhibition induces autophagy and increases cell survival signals^38^. Autophagy can help epithelial cells to eliminate the intracellular GAS that escapes from the endosomes^39^. In addition to GAS, autophagy also targets and traps cellular invasion bacteria such as *Listeria*, *Salmonella* and *Shigella* in non-phagocytic cells^40^. Whether autophagy is involved in GSK-3β inhibition-mediated reduction of the intracellular GAS replication in GAS-infected RAW264.7 cells needs further investigation.

Based on our *in vitro* findings, we point out that GSK-3β inactivation may be an alternative approach to inhibit GAS infection. LiCl, a GSK-3β inhibitor, has been reported to reduce the mortality of the GAS-infected mice through reducing the plasma TNF-α levels^30^, and LiCl also inhibits *K*. *pneumoniae*-induced death and liver injury in mice by decreasing the bacterial burden and cytokine production in blood and liver tissues^41^. Although effects of GSK-3β inactivation on GAS infection with other pathogenic serotypes need further investigation, we suggest GSK-3β inhibitors may be alternative agents to therapy GAS infection.

## Materials and Methods

### Bacterial strains

The wild type GAS strain NZ131 (type M49, T14) was a gift from Dr. D. R. Martin, New Zealand Communicable Disease Center, Porirua. The *sagB*-disrupted mutant of strain NZ131 was generated by plasmid pASC39 insertion, and the *sagB* complementary mutant strain was generated by reintroducing the complementation vector pDL278-*sagB* into the *sagB*-mutant strain as previously reported^19^. The wild type strain GAS was grown in Todd-Hewitt medium supplemented with 0.2% yeast extract (THY) (Difco Laboratories) for 12 h at 37 °C and then subcultured into fresh broth (1:50 [vol/vol]) for another 5 h to get the log-phase bacteria. The *sagB* mutant GAS was grown in THY broth containing 25 μg/ml of chloramphenicol, and the *sagB* complementary mutant strain was grown in THY broth containing 25 μg/ml of chloramphenicol and 100 μg/ml of spectinomycin. The bacterial concentration was determined with a spectrophotometer (Beckman Instruments) by measuring the optical density at 600 nm. To quantitate the exact bacterial concentration, the bacterial suspension was serially diluted with sterile phosphate-buffered saline (PBS), then 0.1 ml was poured on THY agar plates, and incubated at 37 °C overnight.

### Cell culture and bacterial infection

RAW264.7 cells were cultured in DMEM medium supplemented with 5% fetal bovine serum, 100 μg/ml of penicillin and 100 μg/ml of streptomycin (Gibco). Lentiviral-based GSK-3β knockdown in RAW264.7 cells (shGSK-3β-RAW264.7 cells) and the negative control cells (luciferase shRNA, shLuc-RAW264.7 cells), provided by Dr. C. F. Lin, Taiwan, were previously prepared, as reported^42^. RAW264.7, shGSK-3β-RAW264.7, or shLuc-RAW264.7 cells (4 × 10^5^ cells/well in 24-well culture plate), cultured in antibiotic-free DMEM medium containing 5% FBS, were infected with wild type strain GAS, its isogenic mutant or the complementary mutant, which were added in culture medium or within the hanging insert with 0.4 μm membrane (Millipore Corporation), for 1 h at a MOI of 10 or 25. The extracellular GAS were removed by washing twice with PBS and further killed with DMEM containing 50 μg/ml of gentamicin for different periods. In some experiments, cells were pretreated with different concentration of inhibitors, including GSK-3β inhibitor, LiCl, and ROS scavenger, N-acetyl-cysteine (NAC). MG132 and ALLN were used to inhibit proteasome and calpain, respectively. After 1 h treatment with inhibitors, RAW264.7 cells were washed with the antibiotic free DMEM medium and then infected with GAS for 1 h at MOI of 25 as previously described. At different time points, cells were collected, and then examined in different assays.

### ROS production assay

Intracellular ROS levels were assayed by carboxy-H_2_DCFDA reagent or MitoSOX Red reagent (Thermo Fisher). RAW264.7, shLuc-RAW264.7 or shGSK-3β-RAW264.7 cells (2 × 10^5^ cells/well in 96-well culture plate) were loaded with carboxy-H_2_DCFDA or MitoSOX according to the manufacturer’s protocol. Fluorescence of carboxy-H_2_DCFDA was recorded by microplate reader (Life Technologies) at 0~120 min after the bacterial infection, and fluorescence of MitoSOX Red was recorded by microplate reader at 3 h after GAS infection because no notable signal was found before 3 h post-infection. Fluorescence intensity % = (fluorescence of experimental group/fluorescence of medium only group) × 100%.

### Cell death assay

RAW264.7 cells, pretreated with different concentrations of inhibitors, shLuc-RAW264.7 cells or shGSK-3β-RAW264.7 cells were infected with GAS or its isogenic mutants at MOI of 25 as previously described. After cultivation for an additional 18 h with DMEM containing 50 μg/ml of gentamicin, the culture supernatants of the GAS-infected cells and uninfected control group were collected. The levels of lactate dehydrogenase (LDH) released into the cell culture supernatants were measured using the LDH Cytotoxicity Detection Kit (Promega) according to the manufacturer’s instructions, and detected by microplate reader (Life Technologies). The LDH release % = (experimental group-medium background)/(Triton X-100-treated group - medium background) × 100%. The results of three experiments are represented and expressed as the mean ± standard deviation.

### Mitochondrial membrane potential assay

Mitochondrial membrane potential was monitored using the JC1-mitochondrial membrane potential assay kit (Abcam). RAW264.7 cells were loaded with membrane-permeant JC-1 dye 1 h before bacterial infection. According to the infection protocol as described previously, the cells were harvested at different times post-infection. In normal cells with high mitochondrial membrane potential, JC-1 accumulated and aggregated in mitochondria that yielded a red to orange colored emission. In damaged cells, JC-1 accumulated predominantly in cytosol as a monomer, which yielded a green fluorescence, due to low mitochondrial membrane potential. The red and green mean fluorescence intensity (MFI) of JC-1 was recorded by flow cytometric analysis (FACSCalibur, Becton Dickinson). The level of mitochondrial membrane potential changes (Δψm) of RAW264.7 cells was expressed as the ratio of red fluorescent MFI to green fluorescent MFI.

### Western blot analysis

RAW264.7 cells were treated with 10 μM of MG132 or 10 μM of ALLN and then infected with wild type GAS or its isogenic mutant at MOI of 25 as previously described. After cultivation for an additional 2 h with gentamicin–containing DMEM medium, the GAS-infected cells and uninfected control group were collected. Total cell extracts were separated by SDS-PAGE and then transferred to a polyvinylidene difluoride (PVDF) membrane (Millipore Corporation). After 5% skim milk blocking, the PVDF membrane were incubated with rabbit monoclonal antibodies specific for GSK-3β, or phosphor-GSK-3β (Ser9) as primary antibody (Cell Signaling Technology), and mouse anti-GAPDH (Santa Cruz Biotechnology) was used as an internal control. Finally, blots were hybridized with horseradish peroxidase-conjugated goat anti-rabbit IgG (Calbiochem) or anti-mouse IgG (Calbiochem) and developed using enhanced chemiluminescence (Pierce).

### Intracellular bacterial load assay

RAW264.7 cells (4 × 10^5^ cells/well in 24-well culture plate) were infected with wild type strain GAS at MOI of 25 for 1 h in presence of 1,000 μM of LiCl as previously described. Moreover, shGSK-3β-RAW264.7 cells and shLuc-RAW264.7 cells (4 × 10^5^ cells/well in 24-well culture plate) were infected with wild type strain GAS at MOI of 25 for 1 h. After that, gentamicin (50 μg/ml) was added to kill the extracellular GAS for 1 h, and then cells were cultivated in antibiotic-free DMEM medium for an additional 16 h. Total viable bacteria were quantified by plating on THY agar plates as described previously^4^. For examination of initial GAS uptake, gentamicin (50 μg/ml) was added to kill the extracellular GAS for 0.5 h after GAS infection, and then cells were washed, and intracellular bacteria were quantified by plating on THY agar plates as described previously^4^.

### Statistics

The statistical analysis was conducted using Prism 3.0 software (GraphPad Software, San Diego, CA). The data shown in the figures were compared for significance using ANOVA or Student’s *t*-test. Statistical significance was set at *P* < 0.05.

## Supplementary information

Dataset 1

## Data Availability

The datasets generated during and/or analyzed during the current study are available from the corresponding author on reasonable request.
