# Supplementary material for: Streptolysin S induces mitochondrial damage and macrophage death through inhibiting degradation of glycogen synthase kinase-3β in Streptococcus pyogenes infection
Source: Sci Rep. 2019 Mar 29;9:5371. doi: 10.1038/s41598-019-41853-3 (PMC6440947; doi:10.1038/s41598-019-41853-3)
Supplement: Supplementary file 1 — Dataset 1 [file 41598_2019_41853_MOESM1_ESM.pdf]

Streptolysin S induces mitochondrial damage and macrophage death through inhibiting degradation of glycogen synthase kinase-3β in *Streptococcus pyogenes* infection

Nina Tsao, Chih-Feng Kuo, Miao-Hui Cheng, Wei-Chen Lin, Chiou-Feng Lin, & Yee-Shin Lin

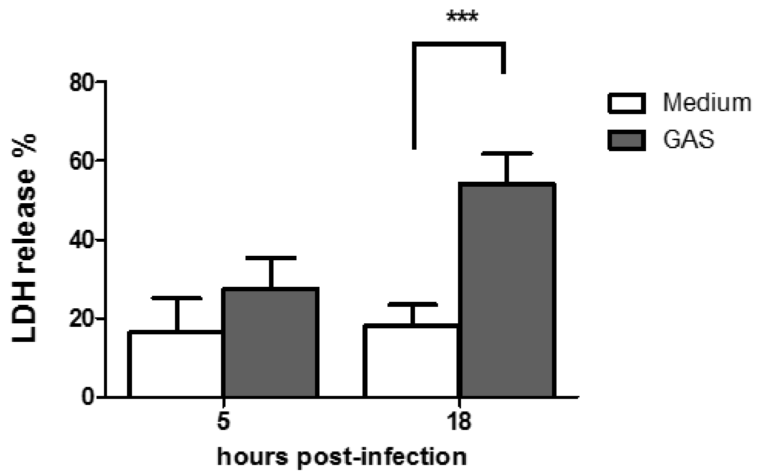

Supplementary Fig. S1. GAS induced cell death of RAW264.7 cells. RAW264.7 cells (4 × 10^5 cells) were infected with the wild type GAS for 1 h at a MOI of 25. The extracellular GAS were removed by washing twice with PBS, and further cultivated cells with DMEM containing 50 µg/ml of gentamicin for 17 h. The culture supernatants of RAW264.7 cells were collected at 18 h post-infection and then measured by LDH detection kit. LDH release % was shown and expressed as the mean ± SD, as described in Materials and Methods. *** $P < 0.001$ compared with medium only group (Two-way ANOVA test followed by Bonferroni test; n = 4).

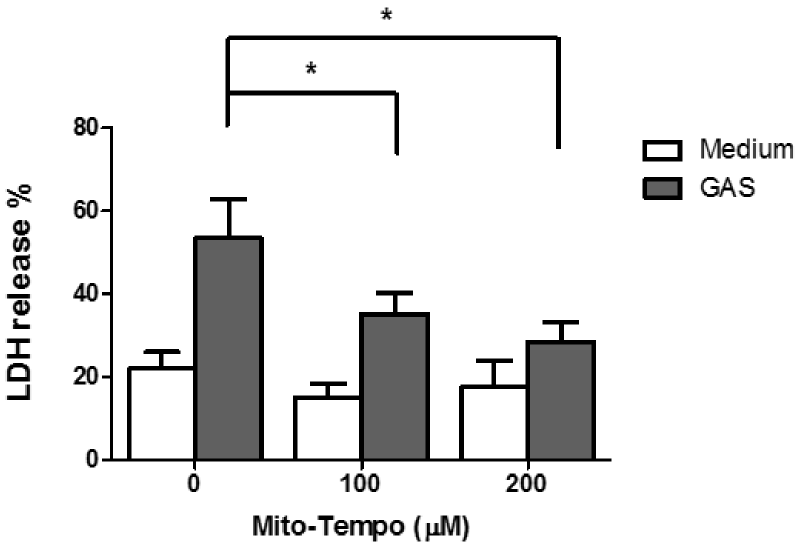

Supplementary Fig. S2. Mito-Tempo increased cell survival of GAS-infected cells. RAW264.7 cells were pretreated with different concentrations of Mito-Tempo for 1 h and then infected with the wild type GAS for 1 h at a MOI of 25. The extracellular GAS were removed by washing twice with PBS, and further cultivated cells with DMEM containing 50 μg/ml of gentamicin and Mito-Tempo for 17 h. The culture supernatants of RAW264.7 cells were collected at 18 h post-infection and then measured by LDH detection kit. LDH release % was shown and expressed as the mean ± SD, as described in Materials and Methods. * $P < 0.05$ compared with GAS-infected group (One-way ANOVA test followed by Tukey's test; n = 4).

a

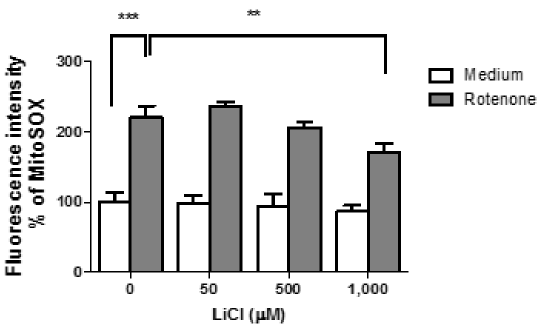

b

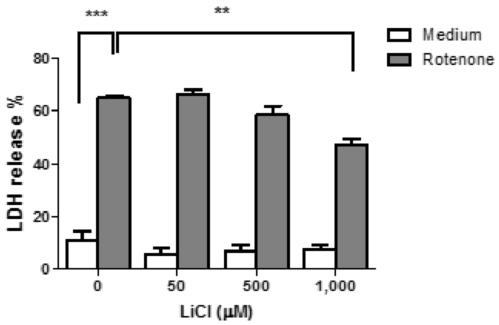

Supplementary Fig. S3. LiCl inhibited rotenone-induced mitochondrial ROS production and cell death. (a) LiCl decreased rotenone-induced mitochondrial ROS production. RAW264.7 cells were pretreated with different concentrations of LiCl for 1 h and then stimulated with rotenone (1 µM) for 5 h. Cells were harvested and mitochondrial ROS of cells were measured by MitoSOX. Fluorescence intensity % was shown and expressed as described in Materials and Methods. Results are represented as mean ± SD. ***$P < 0.001$ compared with medium only group. ** $P < 0.01$ compared with rotenone group (One-way ANOVA test followed by Tukey's test; n = 4). **(b)** LiCl decreased rotenone-induced cell death. RAW264.7 cells were pretreated with different concentrations of LiCl for 1 h and then stimulated with rotenone (1 µM) for 18 h. The culture supernatants of rotenone-treated RAW264.7 cells were collected and then measured by LDH detection kit. LDH release % was shown and expressed as the mean ± SD, as described in Materials and Methods. ***$P < 0.001$ compared with medium only group. ** $P < 0.01$ compared with rotenone group (One-way ANOVA test followed by Tukey's test; n = 4).

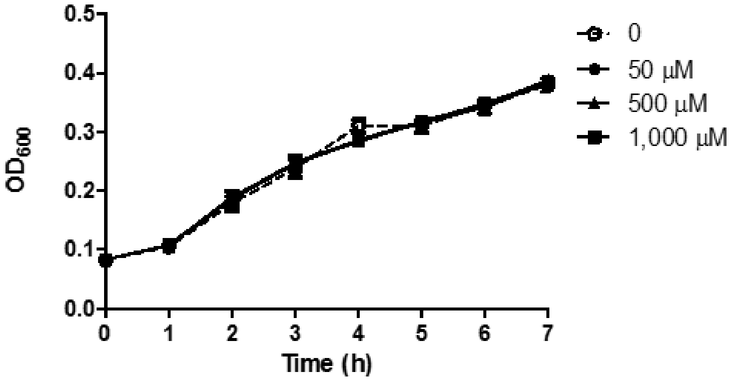

Supplementary Fig. S4. Effect of LiCl on the *in vitro* growth of GAS. GAS NZ131 strain was grown in Todd-Hewitt medium supplemented with 0.2% yeast extract (THY) for 12 h at 37°C and then subcultured into fresh THY broth (1:20 [vol/vol]). After that, the bacterial suspensions were incubated with varying concentrations of LiCl for different amounts of time, and the concentrations of bacteria at each time point were determined with a spectrophotometer by measuring the optical density at 600 nm (OD_{600}).

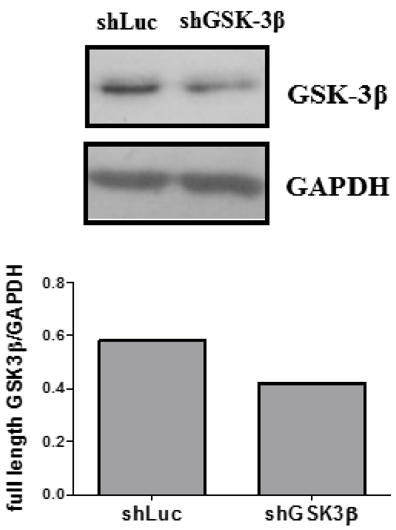

Supplementary Fig. S5. The expression of GSK-3β was silenced in shGSK-3β-RAW264.7 cells. The expression of GSK-3β was silenced in RAW264.7 cells using lentiviral-based shRNA (shGSK-3β) construct, and the luciferase shRNA construct was used as the negative control. The expression of total GSK-3β was determined by Western blotting. GAPDH was used as an internal control. Representative immunoblots from 3 independent experiments were shown. The quantitative ratio of GSK-3β relative to GAPDH was determined by ImageJ.

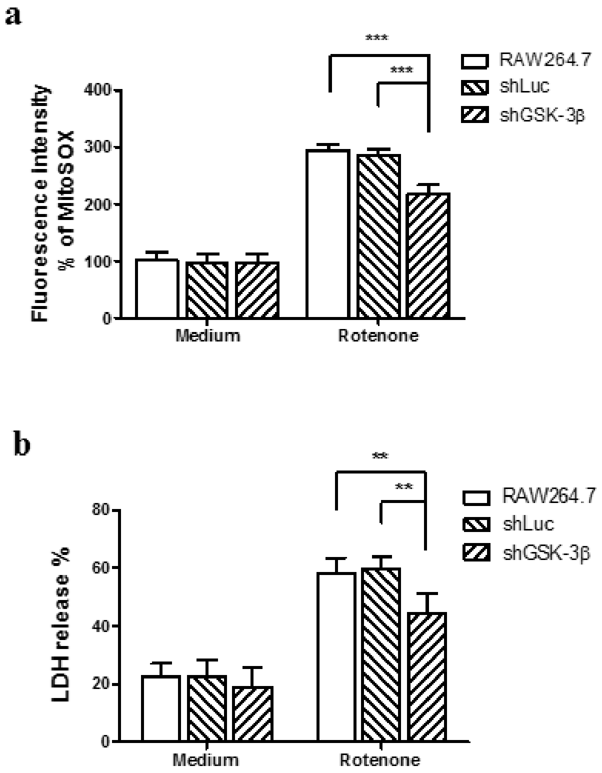

Supplementary Fig. S6. Rotenone-induced mitochondrial ROS production and cell death were inhibited in shGSK-3β-RAW264.7 cells. (a) Knockdown of GSK-3β decreased rotenone-induced mitochondrial ROS production. RAW264.7, shLuc-RAW264.7 or shGSK-3β-RAW264.7 cells (2×10^5 cells/well in 96-well culture plate) were treated with rotenone (1 μM) for 5 h. After that, mitochondrial ROS of cells were measured by MitoSOX. Fluorescence intensity % was shown and expressed as described in Materials and Methods. Results are represented as mean ± SD. ***$P < 0.001$ compared with shGSK-3β-RAW264.7 group versus RAW264.7 group or shLuc-RAW264.7 group (Two-way ANOVA test followed by Bonferroni test; n = 4). **(b)** Knockdown of GSK-3β decreased rotenone-induced cell death. RAW264.7, shLuc-RAW264.7 or shGSK-3β-RAW264.7 cells (2×10^5 cells/well in 96-well culture plate) were treated with rotenone (1 μM) for 18 h. The culture supernatants of rotenone-treated cells were collected and then measured by LDH detection kit. LDH release % was shown and expressed as the mean ± SD, as described in Materials and Methods. **$P < 0.01$ compared with shGSK-3β-RAW264.7 group versus RAW264.7 group or shLuc-RAW264.7 group (Two-way ANOVA test followed by Bonferroni test; n = 4).

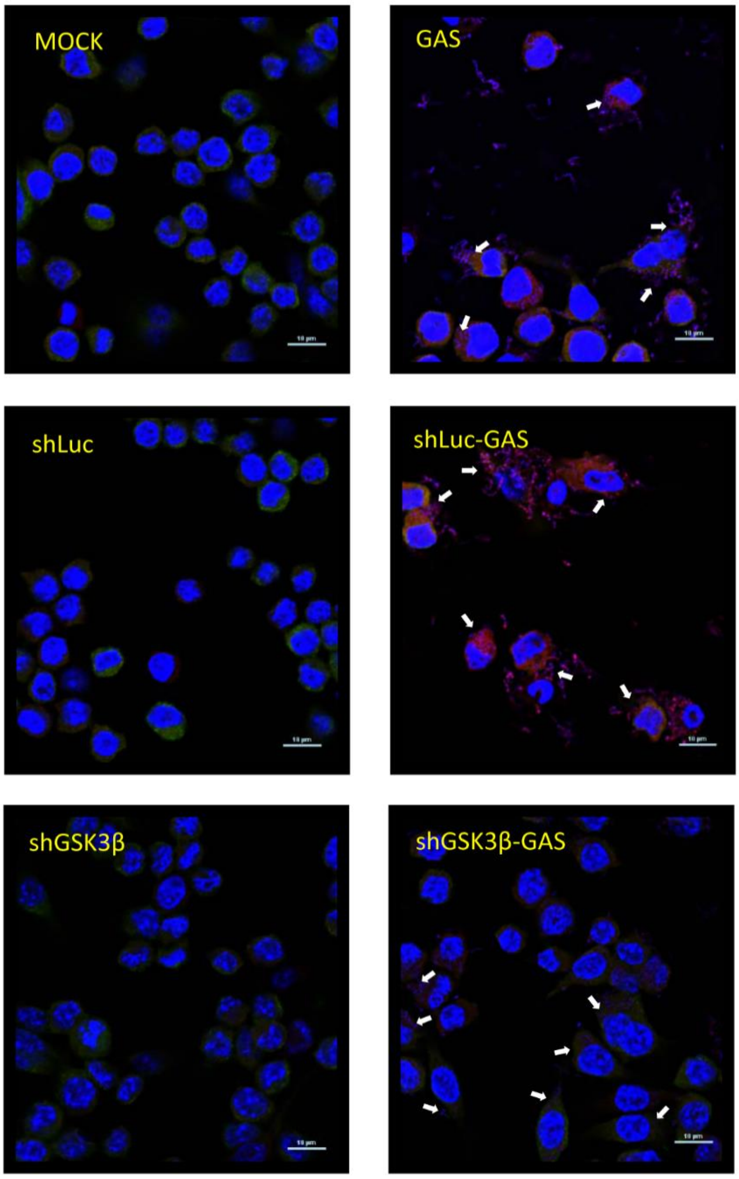

Supplementary Fig. S7. GAS induced mitochondrial ROS production in RAW264.7 cells, shLuc-RAW264.7 cells or shGSK-3β-RAW264.7 cells. RAW264.7, shLuc-RAW264.7 or shGSK-3β-RAW264.7 cells (1×10^6 cells) were infected with the wild type GAS for 1 h at a MOI of 25. The extracellular GAS were removed by washing twice with PBS, and further cultivated cells with DMEM containing 50 μg/ml of gentamicin for 4 h. After that, the cells were fixed with 3.7% formaldehyde for 10 min at room temperature and then stained with 5 μM of MitoSOX at 37℃ for 10 min. After washing cells twice with PBS, the cells were stained with rabbit anti-GSK-3β antibody, followed staining with anti-rabbit IgG conjugated with Alexa 488. Finally, the cells were stained with DAPI and further examined using the confocal microscope. Left three figures represent three kinds of cells without GAS infection. Right three figures represent three kinds of cells with GAS infection. "MOCK" represents RAW264.7 cells without infection. The green color and red color represent GSK-3β staining and MitoSOX staining respectively, while the blue color represents DAPI staining.

The white arrow indicates the clump of intracellular GAS.

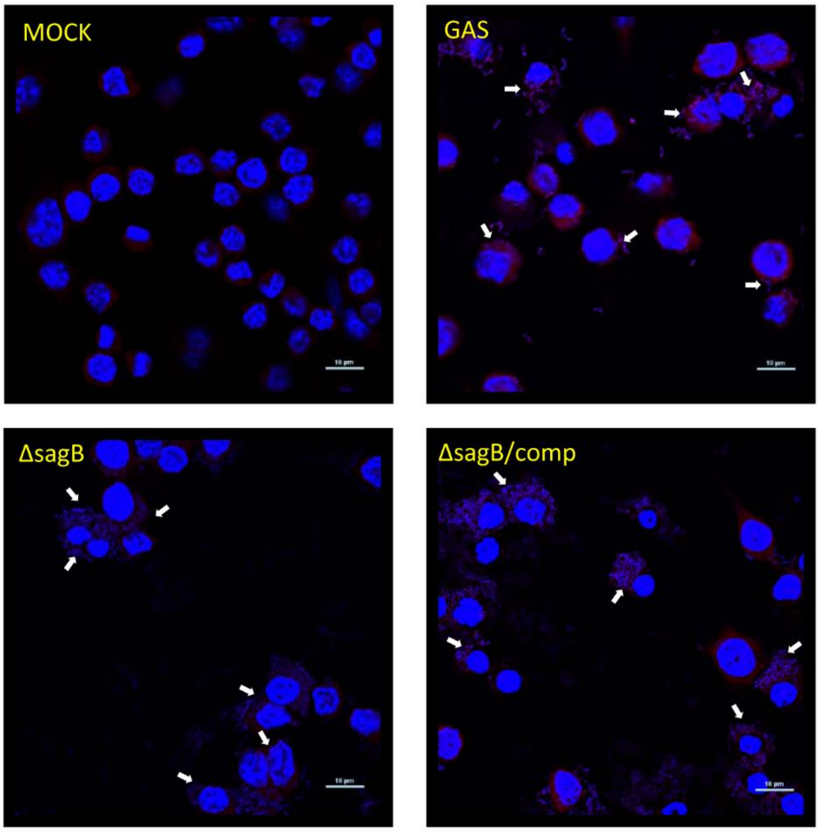

Supplementary Fig. S8. The wild type GAS, its isogenic *sagB* mutant, or *sagB* complementary mutant induced mitochondrial ROS production in RAW264.7 cells. RAW264.7 cells (1×10^6 cells) were infected with the wild type GAS, the *sagB* mutant, or *sagB* complementary mutant for 1 h at a MOI of 25. The extracellular GAS were removed by washing twice with PBS, and further cultivated cells with DMEM containing 50 μg/ml of gentamicin for 4 h. After that, the cells were fixed with 3.7% formaldehyde for 10 min at room temperature and then stained with 5 μM of MitoSOX at 37℃ for 10 min. After washing cells twice with PBS, the cells were stained with DAPI and further examined using the confocal microscope. "MOCK" represents RAW264.7 cells without infection. "GAS" represents the wild type GAS-infected RAW264.7 cells. "△sagB" represents the *sagB* mutant-infected RAW264.7 cells. "△sagB/comp" represents the *sagB* complementary mutant-infected RAW264.7 cells. The blue color and red color represent DAPI staining and MitoSOX staining respectively. The white arrow indicates the clump of intracellular GAS.

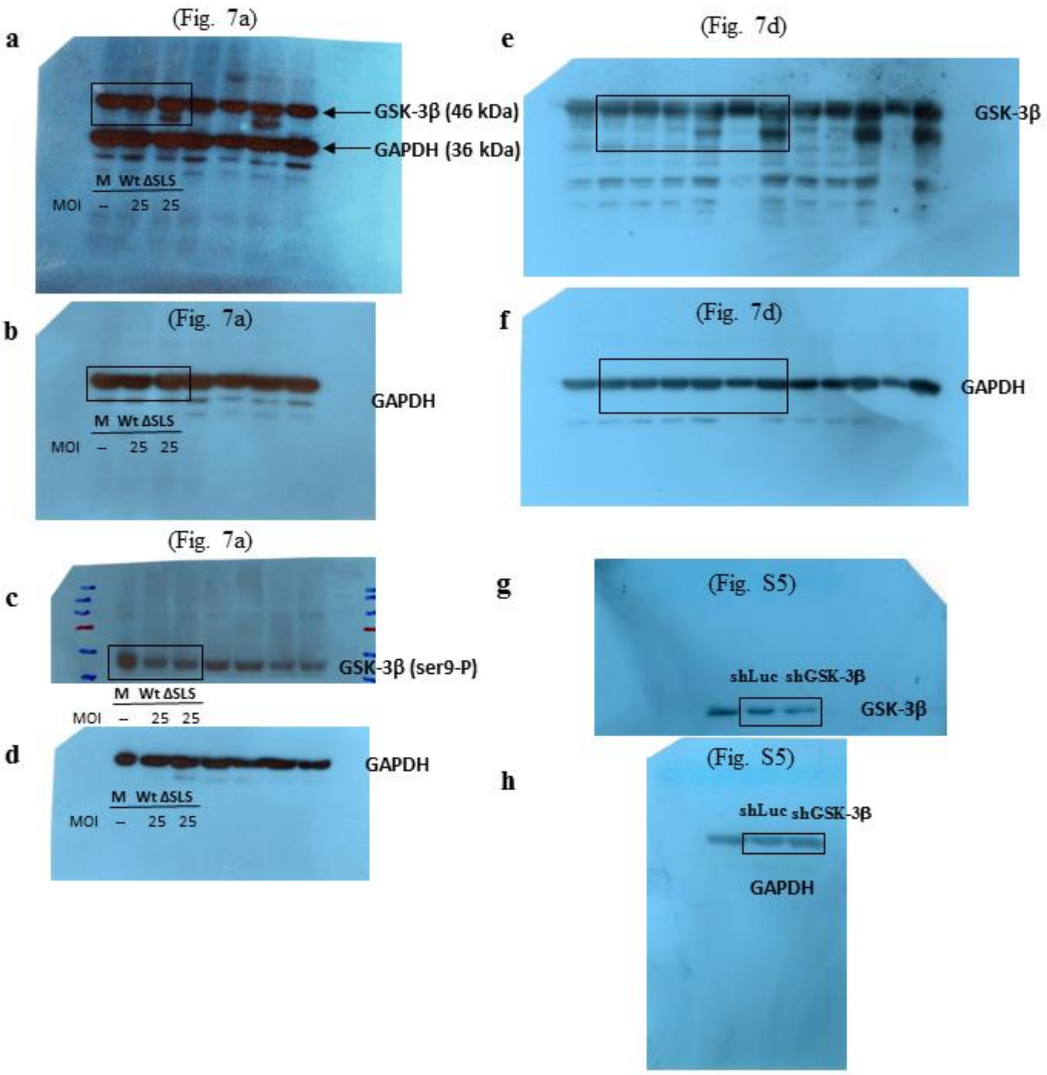

Supplementary Fig. S9. The full sets of data images of immunoblotting analyses for Figure 7 and S5. (a) & (b): The full size images of GSK-3β and GAPDH in Figure 7a. Displayed blots of GSK-3β and GAPDH are cropped from the same gel. The transferred membrane was first blotted with anti-GAPDH antibody **(b)**, and then re-blotted with anti-GSK-3β antibody **(a)**. Hence, there were two kinds of bands (GSK-3β, 46 kDa; GAPDH, 36 kDa) in the Fig. S9a. **(c) & (d):** The full size images of pGSK-3β and GAPDH in Figure 7a. **(e) & (f)**: The full size images of GSK-3β and GAPDH in Figure 7d. **(g) & (h)**: The full size images of GSK-3β and GAPDH in Supplementary Figure S5.
